# Effects of two *Lactobacillus *strains on lipid metabolism and intestinal microflora in rats fed a high-cholesterol diet

**DOI:** 10.1186/1472-6882-11-53

**Published:** 2011-07-03

**Authors:** Ning Xie, Yi Cui, Ya-Ni Yin, Xin Zhao, Jun-Wen Yang, Zheng-Gen Wang, Nian Fu, Yong Tang, Xue-Hong Wang, Xiao-Wei Liu, Chun-Lian Wang, Fang-Gen Lu

**Affiliations:** 1 Department of Gastroenterology, Xiangya Second Hospital, Central South University, Changsha 410011, Hunan Province, PR China; 2 Department of Gastroenterology, Nanhua Second Hospital, Nanhua University, Hengyang 421001, Hunan Province, China; 3 Department of Gastroenterology, Nanhua Hospital, Nanhua University, Hengyang 421002, Hunan Province, China; 4 State Key Laboratory of Medical Genetics, Central South University, Changsha 410011, Hunan Province, China

## Abstract

**Background:**

The hypocholesterolemic effects of lactic acid bacteria (LAB) have now become an area of great interest and controversy for many scientists. In this study, we evaluated the effects of *Lactobacillus plantarum *9-41-A and *Lactobacillus fermentum *M1-16 on body weight, lipid metabolism and intestinal microflora of rats fed a high-cholesterol diet.

**Methods:**

Forty rats were assigned to four groups and fed either a normal or a high-cholesterol diet. The LAB-treated groups received the high-cholesterol diet supplemented with *Lactobacillus plantarum *9-41-A or *Lactobacillus fermentum *M1-16. The rats were sacrificed after a 6-week feeding period. Body weights, visceral organ and fat pad weights, serum and liver cholesterol and lipid levels, and fecal cholesterol and bile acid concentrations were measured. Liver lipid deposition and adipocyte size were evaluated histologically.

**Results:**

Compared with rats fed a high-cholesterol diet but without LAB supplementation, serum total cholesterol, low-density lipoprotein cholesterol and triglycerides levels were significantly decreased in LAB-treated rats (p < 0.05), with no significant change in high-density lipoprotein cholesterol levels. Hepatic cholesterol and triglyceride levels and liver lipid deposition were significantly decreased in the LAB-treated groups (p < 0.05). Accordingly, both fecal cholesterol and bile acids levels were significantly increased after LAB administration (p < 0.05). Intestinal *Lactobacillus *and *Bifidobacterium *colonies were increased while *Escherichia coli *colonies were decreased in the LAB-treated groups. Fecal water content was higher in the LAB-treated groups. Compared with rats fed a high-cholesterol diet, administration of *Lactobacillus plantarum *9-41-A resulted in decreases in the body weight gain, liver and fat pad weight, and adipocytes size (p < 0.05).

**Conclusions:**

This study suggests that LAB supplementation has hypocholesterolemic effects in rats fed a high-cholesterol diet. The ability to lower serum cholesterol varies among LAB strains. Our strains might be able to improve the intestinal microbial balance and potentially improve intestinal transit time. Although the mechanism is largely unknown, *L. plantarum *9-41-A may play a role in fat metabolism.

## Background

Elevated serum cholesterol level is widely recognized as a contributory risk factor for the development of cardiovascular diseases (CVD) such as atherosclerosis, coronary heart disease and stroke. The World Health Organization (WHO) has predicted that by 2030, CVD will remain the leading causes of death and affect approximately 23.6 million people globally [1]. It has been reported that even a 1% reduction in serum cholesterol could reduce the risk of coronary heart disease by 2-3% [2]. Current drug therapies, with their high relative costs and associated side effects, are not viewed to be the optimal long-term answers. The development of alternative management strategies for the treatment of hypercholesterolemia is necessary, especially for people with borderline cholesterol levels.

Since Mann and Spoerry [3] discovered the hypocholesterolemic effects of fermented milk ingested by the Massai tribes people, the relationship between lactic acid bacteria (LAB) and the serum cholesterol has become a focus of great interest. Studies evaluating this relationship have found that lactobacilli or bifidobacteria can exhibit hypocholesterolemic properties in animal models [4-6] and in humans [7-10]. Several hypotheses have been proposed to explain these findings: (1) consumption of cholesterol by intestinal bacteria, thus reducing the amount of cholesterol available for absorption [11,12]; (2) cholesterol may be bound to the bacterial cellular surface [13] or incorporated into the bacterial cellular membranes [14] or converted into coprostanol by cholesterol reductase, which is produced by strains of lactobacilli [15]; (3) inhibition of micelle formation by certain probiotic strains [16]; (4) short-chain fatty acids produced upon selective fermentation of food by intestinal bacterial microflora may lower plasma cholesterol levels [17]; and (5) some bacterial species excrete bile salt hydrolase, leading to increased bile excretion in feces [18]. However, other reports are contradictory and fail to show hypocholesterolemic effects of probiotics [19,20]. Consequently, this area remains controversial. Therefore, more information is required to strengthen the proposed hypotheses and improve our undetstanding of how bacteria affect cholesterol metabolism, which might lead to more appropriate use of probiotics.

To date, most investigations on the effects of LAB on cholesterol have focused on species isolated from dairy products or feces of infants. In our previous work, we isolated several strains of lactobacilli from the stool of healthy adult Chinese volunteers. In *in vitro *screening-experiments, more than 30 strains of the bacteria demostrated a cholesterol-lowering activity. Among these strains, two strains, 9-41A and M1-16 expressed the highest cholesterol-removing abilities in their growth medium and were selected for the present study. We aimed to: 1) examine the effects of these two strains on cholesterol levels, body weight and intestinal microflora in rats; and 2) explore the possible mechanisms by which these strains might exert their hypocholesterolemic effects.

## Methods

### Preparation of bacterial cultures

The two strains used in this study, *Lactobacillus plantarum *9-41-A [GenBank accession no. HQ424577] and *Lactobacillus fermentum *M1-16 [GenBank accession no. HQ423153], were two local strains isolated from the feces of healthy adults and stored at -80°C. They showed good survival at low pH, tolerance to high bile concentration, and abilities to lower cholesterol in *in vitro *trials. After recovery at 35°C for 30 minutes in a biochemical incubator, the two strains were separately inoculated into MRS liquid broth and placed in an anaerobic workstation at 35°C for 24 hours. The strains were harvested by centrifugation at 2000 × g for 20 minutes, washed twice with normal saline (0.9% NaCl), and resuspended at 2 × 10^9 ^CFU/mL in sterile normal saline. Subsequently, 2 mL of the solution was administered intragastrically to the rats daily.

### Animal groups and diets

Forty male Sprague-Dawley rats (conventional clean animal grade), aged 5 weeks and weighing 120.2 ± 4.5 grams, were purchased from Slaccas Laboratory Animal Ltd. (Shanghai, China). All rats were housed individually in metal cages under a controlled room temperatures (20 ± 2°C) and humidity (50 ± 5%), and maintained on a constant 12-hour/12-hour light/dark cycle. The care and use of the animals followed our institutional and national guidelines and all experimental procedures involving animals were approved by the Ethics Committee of the Central South University.

A 1-week adaptive period on a normal diet containing 32% (weight/weight) protein, 5% fat, 2% fiber, 1.8% calcium, 1.2% phosphorus, and a nitrogen-free extract as the remainder (Commercial Chow, Kangqiao Inc., Beijing, China) was instituted [21]. The 40 rats were randomly selected and assigned to four groups of 10 rats each. The initial average body weight was similar among the four groups. The four groups were assigned diets according to the following regimen: (1) control group, normal diet; (2) model group, high-cholesterol diet; (3) *L.9-41-A *group, high-cholesterol diet + *L. plantarum *9-41-A; (4) *L.M1-16 *group, high-cholesterol diet + *L. fermentum *M1-16. The high-cholesterol diet contained 1% (weight/weight) cholesterol, 10% lard, 10% sucrose, 0.3% sodium cholate, 0.2% propylthiouracil and a commercial chow mix (Aoboxing Biotech Co., Ltd., Beijing, China) [21]. Rats had free access to water and their group-specific diet. The *L.9-41-A *group and *L.M1-16 *groups received 2 mL (10^9 ^CFU/mL) daily of *L. plantarum *9-41-A and *L. fermentum *M1-16 solutions respectively, intragastrically for the 6-week study period. Control and model groups received an equivalent amount of normal saline. Body weight was recorded weekly and food consumption was monitored daily. After the feeding period, the rats were fasted for 12 hours and euthanized. The weight of visceral organs (liver, spleen, and kidney) and mesenteric, perirenal, and epididymal white adipose tissues (WAT) were measured.

### Assay for serum lipids

Blood sample (4 mL) was obtained from the celiac vein and transferred to nonheparinized vacuum collection tubes. Tubes were initially held stationary at 0°C for 30 minutes, and then centrifuged at 2000 × g for 15 minutes at 4°C. Serum total cholesterol (TCH), high-density lipoprotein cholesterol (HDL-C), low-density lipoprotein cholesterol (LDL-C) and triglycerides (TG) were measured with commercial kits (Daiichi Chemicals Co., Ltd, Tokyo, Japan) and a chemical analyzer 7020 (Hitachi, Tokyo, Japan). Atherogenic indexes (TCH/HDL-C and TG/HDL-C ratios) were then calculated.

### Assay for liver TCH, TG and fecal sterol contents

After euthanasia, rat livers were removed, rinsed with physiological saline solution, blotted dry and weighed. The middle lobe was standardized as the sampling region. A portion of the tissues was sectioned and soaked in 10% (volume/volume) formaldehyde for 24 hours for subsequent staining, with the remainder stored at -80°C for further testing. Liver TCH and TG contents were determined after homogenization with Folch solution(chloroform/methanol ratio = 2:1) [22].

Fecal droppings were collected during the last 3 days of life, and fecal neutral and acidic sterols were extracted using Tokunaga' s method [23]. A sample (1 gram) of wet feces was extracted with 10 mL of acetone/ethanol (1:1) mixture. After drying, 3 mL of diethyl ether and 3 mL of 1 N NaOH solution were added to separate the neutral and acidic sterols. The ether phase containing neutral sterols was dried and dissolved in 3 mL of acetic acid for cholesterol measurement. The NaOH solution phase was used for bile acid measurement with a commercial kit (Daiichi Chemicals Co., Ltd., Tokyo, Japan).

### Histopathology of liver and adipocytes in the WAT

Liver samples from each rat were fixed in 10% (volume/volume) formaldehyde, embedded in paraffin and stained with hematoxylin-eosin (HE). The perirenal WAT was removed and embedded after delipidation. After fixation in 20% (v/v) formalin/1% glutaraldehyde, 3-mm tissue sections were created and stained with HE [24]. We took three samples from liver and the WAT of each rat, and made three replicates for each sample. All histologic section was observed under 5 high-power fields. The classification and degree of fatty deposition used was as follows [22]: +, mild fatty degeneration, fatty hepatocytes occupy 30%-50% of the hepatic parenchyma; ++, moderate fatty degeneration, fatty hepatocytes occupy 50%-75% of the hepatic parenchyma; +++, severe fatty degeneration fatty hepatocytes occupy > 75% of the hepatic parenchyma. The number and size of the adipocytes in the WAT were measured using an ocular micrometer (YanFeng Instrument Co., Ltd., Hengyang, China).

### Analysis of intestinal microflora

The cecum and a portion of the adjacent colon tissue of each rat were removed and placed in capped sterile tubes. After transfer to a laminar flow cabinet, 1 gram of each sample was transferred to a tube with 9 mL of 0.9% NaCl solution and homogenized by vortexing for 10 minutes. Ten fold serial dilutions of each sample were performed to obtain 10-μL concentrations, which were plated with selective culture medium in triplicate. EMB agar (pH 7.1 ± 0.2) was used for *Escherichia coli (E. coli)*, TCH sodium azide agar (pH 7.3 ± 0.1) was used for *Enterococcus faecalis*, LBS agar (pH 5.5 ± 0.2) was used for LAB, and BS agar (pH 7.2 ± 0.1) was used for bifidobacteria. All media were obtained from Hope Bio-Technology (Qingdao, China). Aerobic plates were placed in a biochemistry incubator at 37°C for 24 hours while anaerobic plates incubated at 37°C for 48 hours. The colonies counted after incubation represented the numbers of *E. coli, Enterococcus faecalis, Lactobacillus*, and *Bifidobacterium*.

### Measurement of fecal water content

In the middle of the third and sixth weeks, rat feces were collected, weighed and dried at 80°C in a DZF-6050 vacuum drying oven (MingKe Instrument Co., Ltd., Shengzhen, China). until a constant weight was achieved within 24 hours, and then reweighed. Fecal water content was calculated as follows: fecal water content (%) = [(weight before drying-weight after drying)/weight before drying] × 100.

### Statistical Analysis

All data was analysed in duplicate with SPSS 15.0 software (SPSS Inc., Chicago, IL, USA). One-way analysis of variance (ANOVA) with Duncan's multiple range test was performed to compare any significant differences (p < 0.05) in the variables between groups. Experimental data were presented as the mean ± standard deviations (SD) of the mean. Rank and frequency data were analyzed by a nonparametric test.

## Results

### Growth of rats

All the rats appeared healthy throughout the feeding period. There were no significant differences in total food intake (p > 0.05) among the four groups. All rats fed the high-cholesterol diet exhibited higher (p < 0.05) body weight gains and food efficiencies than control rats (Table 1). The *L*.9-41-A group showed less weight gain (a reduction of 13.3%, p < 0.05) and food efficiency than the model group while the *L*.M1-16 group had no obvious difference in weight gain and food efficiency compared with the model group.

**Table 1:** Body weight gain, total food intake, and food efficiency (n = 10 per group) after 6 weeks

Group	Body weight gain(g)	Total food intake(g)	Food efficiency*(%)
Control	144.6^c ^± 10.2	879.9^a ^± 18.9	16.4^c ^± 1.1
Model	177.0^a ^± 8.8	875.1^a ^± 25.6	20.2^a ^± 1.0
*L*. 9-41-A	153.5^b ^± 8.2	881.3^a ^± 21.2	17.4^b ^± 1.0
*L*.M1-16	172.4^a ^± 10.4	883.9^a ^± 22.7	19.50^a ^± 1.0

Control group: normal diet; model group: high-cholesterol diet; *L*.9-41-A group: high-cholesterol diet + *L. plantarum *9-41-A; *L*.M1-16 group: high-cholesterol diet+ *L. fermentum *M1-16.

*Food efficiency (%) = (body weight gain/food intake) × 100.

The data are shown as the mean ± standard deviation ^a,b,c ^Mean values within a column with different superscript letters differ significantly (p < 0.05, n = 10).

### Blood lipid analyses

**Figure 1:**
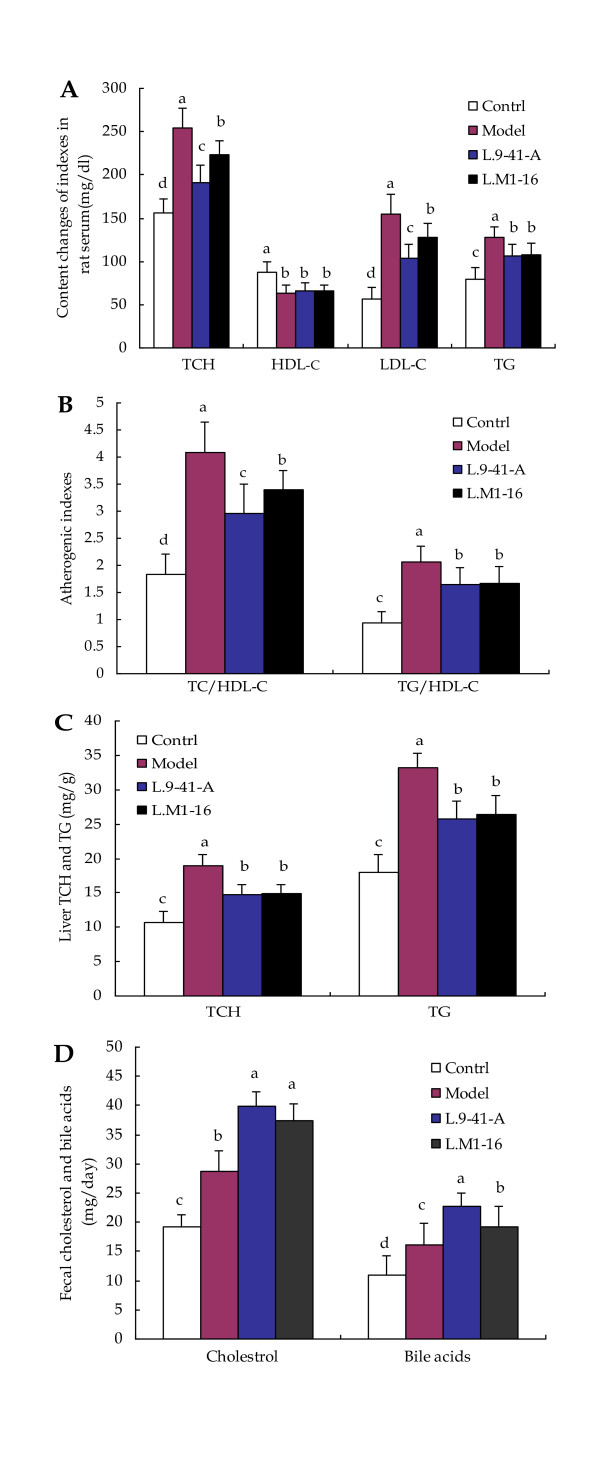
**Effects of the two LAB strains on lipid metabolism in rats fed a high-cholesterol diet**. **A: **Serum TCH, HDL-C, LDL-C and TG contents; **B: **Atherogenic indexes of the rats; **C: **Liver TCH and TG contents of the rats; **D: **Fecal total cholesterol and bile acid content. The data are shown as the mean ± standard deviation (n = 10), ^a,b,c,d ^Mean values in each panel with different superscript letters differ significantly (p < 0.05).

Serum TCH, HDL-C, LDL-C and TG levels in the four groups were shown in Figure 1A. The TCH and LDL-C levels differed significantly among the four groups. Rats fed a high-cholesterol diet had greatly increased levels of serum TCH, LDL-C and TG levels compared with rats fed the normal diet (p < 0.05). Compared with the control group, the model group showed a 63.7% increase in TCH (254.89 ± 22.47 *vs*. 155.72 ± 15.96 mg/dL, p < 0.05), a 176.6% increase in LDL-C (155.16 ± 22.43 *vs*. 56.10 ± 14.03 mg/dl, p < 0.05) and a 60.3% increase in TG (128.31 ± 12.05 *vs*. 80.03 ± 12.39 mg/dL, p < 0.05). The two LAB strains displayed *in vivo *hypocholesterolemic abilities. Compared with the model group, the *L*.9-41-A group achieved a maximal TCH reduction of 25.3% (190.36 ± 20.67 *vs*. 254.89 ± 22.47 mg/dL, p < 0.05) and an LDL-C reduction of 32.9% (104.13 ± 16.11 *vs*. 155.16 ± 22.43 mg/dL, p < 0.05) while the *L*.M1-16 group achieved a TCH reduction of 12.5% (223.12 ± 16.84 *vs*. 254.89 ± 22.47 mg/dL, p < 0.05) and an LDL-C reduction of 17.3% (128.28 ± 15.43 mg/dL *vs*. 155.16 ± 22.43 mg/dL, p < 0.05). The control group showed the highest HDL-C concentration, and the two LAB strains did not demonstrate obvious influences on the HDL-C levels. However, serum TG level was significantly lower in the LAB-treated groups than in the model group. Specifically, the *L*.9-41-A group displayed a TG reduction of 16.9% (106.65 ± 12.58 *vs*. 128.31 ± 12.05 mg/dL, p < 0.05) while the *L*.M1-16 group displayed a TG reduction of 15.7% (108.12 ± 13.35 *vs*. 128.31 ± 12.05 mg/dL, p < 0.05), while the two LAB-treated groups showed no significant difference (p > 0.05). The control group had the lowest (p < 0.05) atherogenic indexes among all four groups. Compared with the model group, the atherogenic indexes were significantly lower in the *L*.9-41-A group (TCH/HDL-C ratio, 2.95 ± 0.54 *vs*. 4.08 ± 0.57; TG/HDL-C ratio, 1.65 ± 0.30 *vs*. 2.06 ± 0.29) and the *L*.M1-16 group (TCH/HDL-C ratio 3.40 ± 0.36 *vs*. 4.08 ± 0.57; TG/HDL-C ratio, 1.66 ± 0.32 *vs*. 2.06 ± 0.29) (Figure 1B). Between the two LAB-treated groups, the *L*.9-41-A group had lower (p < 0.05) TCH/HDL-C ratio than the *L*.M1-16 group, and the TG/HDL-C ratio was similar between the two groups (p > 0.05).

### Liver and fecal lipid analyses

Liver and fecal lipid contents were shown in Figures 1C and 1D. The control group displayed the lowest levels of both hepatic TCH (10.69 ± 1.54 mg/g) and TG (17.90 ± 2.67 mg/g). The *L*.9-41-A and *L*.M1-16 groups showed no obvious differences in both hepatic TCH and TG levels, but they all had lower concentrations (p < 0.05) than the model group (TCH, 14.70 ± 1.42 and 14.82 ± 1.31 *vs*. 18.93 ± 1.59 mg/g, respectively; TG, 25.82 ± 2.50 and 26.44 ± 2.68 *vs*. 33.14 ± 2.19 mg/g, respectively). The high-cholesterol diet increased the fecal excretion of cholesterol and bile acids. Compared with the model group, the *L*.9-41-A group displayed a 39.2% increase in fecal cholesterol (39.94 ± 2.39 *vs*. 28.68 ± 3.48 mg/day, p < 0.05) and a 40% increase in fecal bile acids (22.70 ± 2.29 *vs*. 16.21 ± 3.68 mg/day, p < 0.05) while the *L*.M1-16 group showed a 30.2% increase in fecal cholesterol (37.33 ± 2.92 *vs*. 28.68 ± 3.48 mg/day, p < 0.05) and a 19% increase in fecal bile acids (19.29 ± 3.41 *vs*. 16.21 ± 3.68 mg/day, p < 0.05). The two LAB-treated groups had similar amount of fecal cholesterol content but the *L*.9-41-A group had more fecal bile acids (p < 0.05) than the *L*.M1-16 group.

### Visceral organ and WAT weight

Visceral organ and WAT weight were shown in Table 2. The rats in the control group had the lowest liver and WAT weight. The rats in the *L*.9-41-A and *L*.M1-16 groups had significantly lower liver weight than the rats in the model group (p < 0.05). There were no significant differences in the weight of spleens or kidneys among the four groups. Control group had the lowest WAT weight in all four gourps. Compared with the model group, WAT weights extracted from the rats of *L*.9-41-A group was dramatically reduced (p < 0.05), while the *L*.M1-16 group rats only showed a reduction in the weight of the epididymal fat pad (p < 0.05). Rats of the *L*.9-41-A group also showed smaller weight of mesenteric and perirenal fat pad than rats of the *L*.M1-16 group.

**Table 2:** Effects of *L. plantarum *9-41-A and *L. fermentum *M1-16 on organ and WAT weight in rats

Weight index(g)	Group (mean ± SD, n = 10 per group)			
	
	Control	Model	*L*.9-41-A	*L*.M1-16
Liver	9.5^c ^± 0.8	12.5^a ^± 1.2	10.3^b ^± 1.1	10.7^b ^± 1.4
Spleen	0.6 ± 0.1	0.6 ± 0.1	0.6 ± 0.1	0.6 ± 0.1
Kidney	1.7 ± 0.1	1.7 ± 0.1	1.7 ± 0.1	1.7 ± 0.1
MFP	4.0^b ^± 0.4	5.0^a ^± 0.8	4.1^b ^± 0.9	4.8^a ^± 0.6
PFP	3.5^c ^± 0.6	6.2^a ^± 1.8	4.9^b ^± 1.0	6.1^a ^± 1.0
EFP	4.0^c ^± 0.4	5.4^a ^± 1.3	4.5^b ^± 1.0	4.9^b ^± 1.2

Control group: normal diet; model group: high-cholesterol diet; *L*.9-41-A group: high-cholesterol diet + *L. plantarum *9-41-A; *L*.M1-16 group: high-cholesterol diet+ *L. fermentum *M1-16.

MFP, mesenteric fat pad; PFP, perirenal fat pad; EFPs, epididymal fat pad

The data are shown as the mean ± standard deviation ^a,b,c ^Mean values within a row with different superscript letters differ significantly (p < 0.05, n = 10).

### Hepatic lipid deposition in the four groups

**Figure 2:**
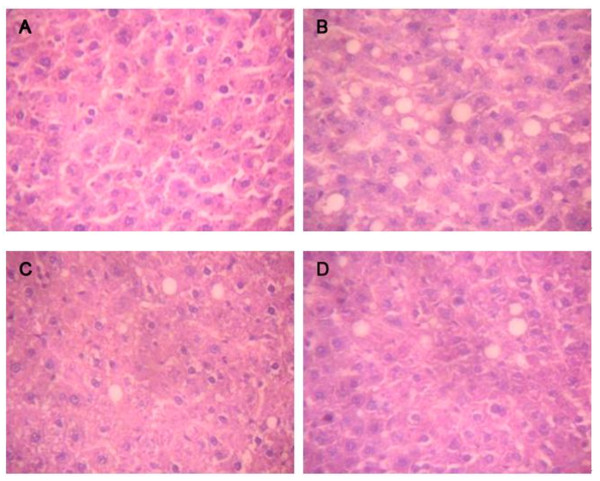
**Histology of liver steatosis in the four groups**. A: normal diet; B: high-cholesterol diet; C: high-cholesterol diet + *L. plantarum *9-41-A; D: high-cholesterol diet+ *L. fermentum *M1-16. All the photomicrographs show HE staining (original magnification × 400).

The LAB-treated rats exhibited an overall normal gross liver appearance. Liver lipid deposition was evaluated in a semiquantitative manner. As shown in Figure 2, no fatty vacuolization was found in the control group. The liver tissue in the model group had a moderate degree of vacuolization and increased lipid deposition in the cytoplasm, which was obviously lower in the *L*.9-41-A and *L*.M1-16 groups. Hepatocyte steatosis was alleviated in the control, *L*.9-41-A and *L*.M1-16 groups compared with the model group by a non-parametric test (χ2 = 24.118, p < 0.001) (Figure 2 and Table 3).

**Table 3:** Degrees of liver lipid deposition

Group	Degree of fatty deposition* (n = 10 per group)			
	
	-	+	++	+++
Control	10	0	0	0
Model	0	6	4	0
*L*.9-41-A	8	2	0	0
*L*.M1-16	7	2	1	0

Control group: normal diet; model group: high-cholesterol diet; *L*.9-41-A group: high-cholesterol diet + *L. plantarum *9-41-A; *L*.M1-16 group: high-cholesterol diet+ *L. fermentum *M1-16.

*Standard of classification and score for the degree of lipid deposition: +, mild fatty degeneration, fatty hepatocytes occupy 30-50% of the hepatic parenchyma; ++, moderate fatty degeneration, fatty hepatocytes occupy 50-75% of the hepatic parenchyma; +++, severe fatty degeneration, fatty hepatocytes occupy > 75% of the hepatic parenchyma.

### Effects of *L. plantarum *9-41-A and L. *fermentum *M1-16 on adipocyte numbers and sizes in the WAT

**Figure 3:**
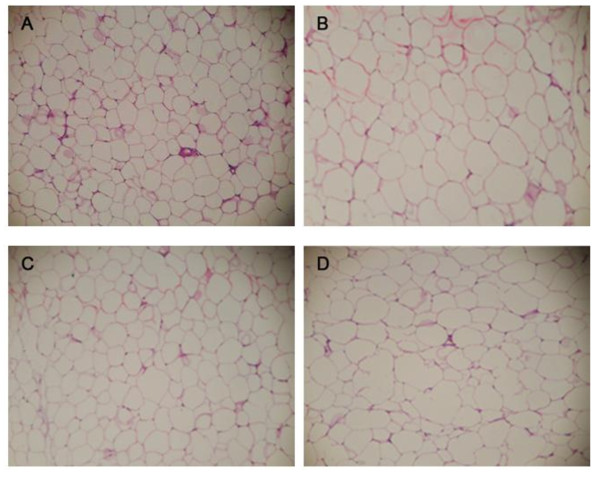
**Adipocytes in WAT sections in the four groups**. A: normal diet; B:high-cholesterol diet; C: high-cholesterol diet + *L. plantarum *9-41-A; D: high-cholesterol diet + *L. fermentum *M1-16. All the photomicrographs show HE staining (original magnification × 400).

Morphometric analyses of the WAT were shown in Figure 3 and Table 4. The perirenal WAT in the model group rats had approximately half the numbers of adipocytes observed in the control and *L*.9-41-A groups. The *L*.M1-16 group had more adipocytes under each field of view than the model group, but the number was less than the control and *L*.9-41-A groups (p < 0.05). The average adipocyte size in the model group was significantly (p < 0.05) larger than that in the control and *L*.9-41-A groups. The *L*.M1-16 group showed a smaller adipocyte size than that in the model group, although this was not significant.

**Table 4:** Adipocyte number and size in the WAT under each field (n = 10 per group)

Group	Number/HP	Major axis (μm)	Minor axis (μm)	Diameter (μm)
Control	56.4^a ^± 4.2	36.6^b ^± 3.6	31.5^b ^± 3.2	33.8^b ^± 3.3
Model	27.5^c ^± 4.3	55.2^a ^± 4.4	49.19^a ^± 5.6	52.2^a ^± 5.0
*L*.9-41-A	50.3^a ^± 5.7	39.4^b ^± 3.9	36.7^b ^± 2.3	38.0^b ^± 3.0
*L*.M1-16	39.1^b ^± 6.3	51.9^a ^± 7.3	43.75^a ^± 5.9	47.8^a ^± 6.6

Control group: normal diet; model group: high-cholesterol diet; *L*.9-41-A group: high-cholesterol diet + *L. plantarum *9-41-A; *L*.M1-16 group: high-cholesterol diet + *L. fermentum *M1-16.

Diameter = (major axis+minor axis)/2.

The data are shown as the mean ± standard deviation. ^a,b,c ^Mean values within a column with different superscript letters differ significantly (p < 0.05, n = 10).

### Microbial populations

**Figure 4:**
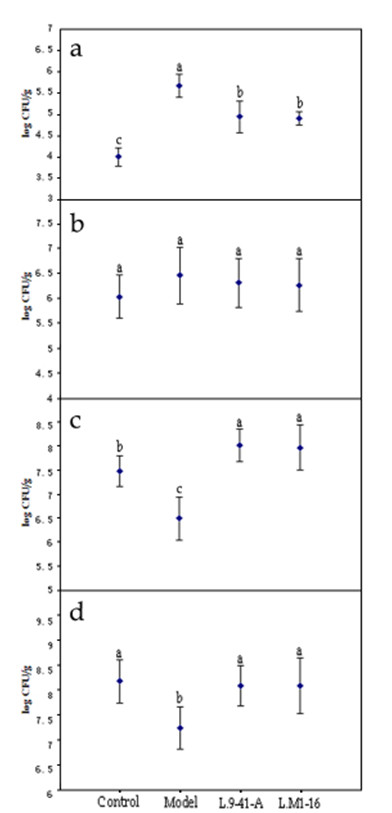
**Populations of *E. coli, Enterococcus faecalis, Lactobacillus*, and *Bifidobacterium *in rat cecums in the four groups**. Control group: normal diet; model group: high-cholesterol diet; *L*.9-41-A group: high-cholesterol diet + *L. plantarum *9-41-A; *L*.M1-16 group: high-cholesterol diet + *L. fermentum *M1-16. a: Counts of *E. coli *colonies. b: Counts of *Enterococcus faecalis *colonies. c: Counts of *Lactobacillus *colonies. d: Counts of *Bifidobacterium *colonies. ^a,b,c ^Mean values with different superscript letters differ significantly (*p *< 0.05).

Figure 4 showed the effects of the different diets and LAB supplementation on the rat intestinal bacteria flora. The model group showed the highest (p < 0.05) total *E. coli *content (5.7 log CFU/g), followed by the *L*.9-41-A group (4.9 log CFU/g), *L*.M1-16 group (4.8 log CFU/g) and control group (4.0 log CFU/g). There were no significant differences in the *Enterococcus faecalis *colonies among the four groups. Compared with the model group (6.5 log CFU/g for *Lactobacillus *and 7.2 log CFU/g for *Bifidobacterium*), significant increases (p < 0.05) in the *Lactobacillus *and *Bifidobacterium *colonies were observed in the cecal samples from the control group (7.5 log CFU/g for *Lactobacillus *and 8.1 log CFU/g for *Bifidobacterium*), *L*.9-41-A group (8.1 log CFU/g for *Lactobacillus *and 8.0 log CFU/g for *Bifidobacterium*) and *L*.M1-16 group (7.9 log CFU/g for *Lactobacillus *and 8.0 log CFU/g for *Bifidobacterium*). There were no significant differences (p > 0.05) in the number of *Lactobacillus *and *Bifidobacterium *colonies between the *L*.9-41-A group and the *L*.M1-16 group, and the two groups had more *Lactobacillus *colonies than the control group (p < 0.05).

### Fecal water content

**Figure 5:**
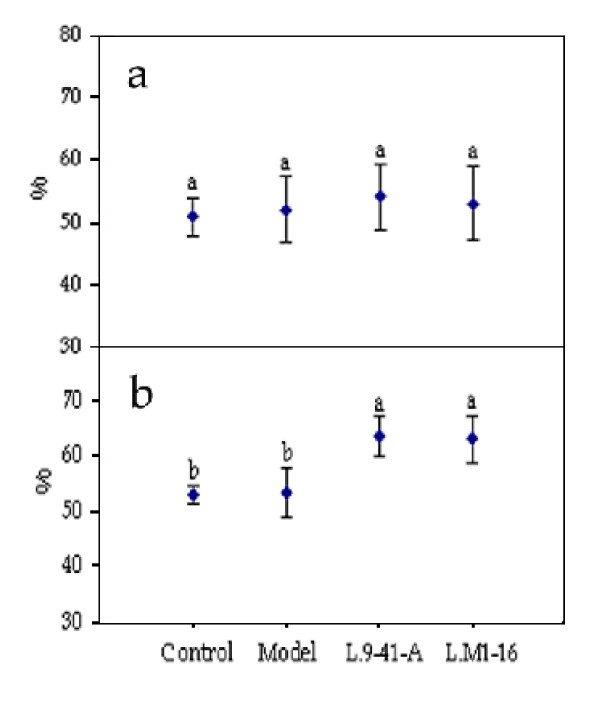
**Fecal water content from the rats in the four groups**. Control group: nomal diet; model group:high-cholesterol diet; *L*.9-41-A group:high-cholesterol diet + *L. plantarum *9-41-A; *L.M1*-16 group:high-cholesterol diet + *L. fermentum *M1-16. a:Water content on the middleday of the third week. b:Water content on the middleday of the sixth week. ^a,b ^Mean values with different superscript letters differ significantly (p < 0.05).

Fecal water content data collected in the middle of the third and sixth weeks was shown in Figure 5. The fecal water content showed no significant differences in the third week among the four groups (range, 50-55%, p > 0.05). However, at the end of the feeding period, there was a higher fecal moisture content in the *L*.9-41-A (63%) and *L*.M1-16 (62%) groups than in the control (52%) and model (53%) groups.

## Discussion

Elevated serum cholesterol and LDL-C are strongly associated with increasing CVD risks. Recent reports demonstrating the hypocholesterolemic effects of probiotics have led to increased interest in this treatment modality, which is less expensive and could be considered a "natural health remedy". We tested two fecal-derived strains of LAB and found the hypocholesterolemic effects *in vivo*. Supplementation with either of the two strains was effective in reducing serum cholesterol, LDL-C and TG concentrations in rats compared to those fed the same high-cholesterol diet but without LAB supplementation. In particular, these effects were more evident with *L. plantarum *9-41-A (TCH, TG and liver cholesterol reduced by 25.3%, 16.9% and 22.3%, respectively). Our results confirm those described in previous reports [6,21,25]. However, there are also differing reports for *Lactobacillus rhamnosus, L. fermentum *and *Lactobacillus acidophilus *[19,20,26]. These conflicting findings could be explained by confounding variables, such as different sources and properties of LAB strains, varying clinical characteristics of the subjects, LAB doses, and lengths of treatment[21,27].

LDL-C is the main component of serum cholesterol. Therefore, lowering of the LDL-C level may be an important factor for reducing serum total cholesterol. Our two LAB strains reduced the higher serum LDL-C concentrations in rats caused by the high-cholesterol diet compared with the model group. Park *et al. *[28] had reported that the hepatic LDL-R mRNA expression of rats fed on either a normal diet or a high-cholesterol diet increased with L. acidophilus ATCC 43121 supplementation and the serum LDL-C level reduced accordingly, but by what means the LAB affect the hepatic LDL-R mRNA expression needs to be investigated furtherly. The two strains did not appear to affect HDL-C concentrations. Ibrahim *et al. *[29] and St-Onge *et al. *[30] reported similar results about the influence of fermented milk on the HDL-C level in rats or humans. However, Hashimoto *et al. *[31] found that a diet containing *Lactobacillus casei *TMC 0409 could increase the concentration of HDL-C in rats. Shin *et al. *[32] also recently reported that the serum HDL-C levels in rats were increased by sonication-killed *Bifidobacterium longum*. A convincing explanation for these controversial findings remains unclear, although different properties of the bacterial strains could be considered. We also found that the TCH/HDL-C and TG/HDL-C ratios, which are highly independent predictors of CVD [33,34], were reduced in rats supplemented with LAB. Further studies are necessary to confirm these observations, and thereby more definitively predict whether LAB supplementation does reduce the risk of CVD.

As expected, we confirmed that the high-cholesterol diet increased hepatic cholesterol and TG content in rats. Rats supplemented with *L. plantarum *9-41-A and *L. fermentum *M1-16 displayed significant reductions in hepatic cholesterol and TG levels and lipid deposition. These findings demonstrated that the serum cholesterol and TG levles in LAB-treated rats were actually reduced, rather than merely being redistributed from the blood to the liver. The lower level of hepatic cholesterol and TG content could decrease the conversion of IDL to LDL-C particles, and result in the decrease of serum LDL-C concentration, that might be another reason for the effects of our two strains to lower LDL-C level.

It has been reported that certain probiotic strains could enhance fecal elimination of bile acids and this may alter the cholesterol synthesis pathways and resulted in a decrease of serum cholesterol concentration [28]. We found more bile acids in the feces of the LAB-treated rats, especially in those rats in the *L*.9-41-A group. Therefore, one possible mechanism for how our two strains lowered the cholesterol levels may be that more bile acids in the enterohepatic circulation were deconjugated and precipitated, and excreted from the feces, resulting in reduced serum cholesterol concentrations. This may partly explain that why rats in the *L*.9-41-A group had lower serum cholesterol level than rats in the *L*.M1-16 group. In addition, more fecal cholesterol was detected in the *L*.9-41-A and *L.M1-16 *group rats. LAB may assimilate dietary cholesterol by incorporating it into their cellular membranes or cell walls, and then via fecal excretion [35]. Thus, LAB strains with hypocholesterolemic properties may lower serum cholesterol in multiple ways.

Generally, a high-cholesterol diet could increase the body weight. To the best of our knowledge, only a few studies have reported a weight-lowering effect of LAB besides a hypocholesterolemic ability. We observed significant weight loss (p < 0.05) in the rats supplemented with *L. plantarum *9-41-A compared with the model group rats, and decreased liver and adipose tissue weight contributed to this finding. In addition, adipocytes of the mesenteric WAT in the *L*.9-41-A rats were smaller than those in the model group rats. Some bacterial strains are considered to have preventive effects on excessive body weight gain[22,36,37]. Fat digestion and absorption in the small intestine may be affected by the gut microbiota. Hamad *et al. *[36] reported a reduction in the adipocyte size of rats treated with fermented skimmed milk containing *Lactobacillus gasseri *SBT2055. The authors therefore considered that the skimmed milk fermented by *L. gasseri *SBT2055 would reduce fat storage through the inhibition of dietary fat absorption. Increased bile acid excretion in the feces could elevate bile acid synthesis, and this process may also contribute to the observed weight-lowering effect. Ikemoto *et al. *[38] found that cholate could inhibit diet-induced obesity by decreasing acyl-CoA synthetase mRNA expression. Watanabe *et al. *[39] also reported that bile acids could induce energy expenditure. Nevertheless, additional research on the mechanisms behind these actions is required.

Gut microbiota distortions may potentially contribute to a wide range of diseases. Effective interventions in the gut microbiota composition with probiotic supplementation may improve health and prevent the onset of certain diseases[40]. Analyses of the rat intestinal microflora demonstrated that *E. coli *was increased while *Lactobacillus *and *Bifidobacterium *were decreased in the model group, implying that the high-cholesterol diet may have interfered with the intestinal microbiota. Stepankova et al. [41] found that absence of gut microbiota (germ-free conditions) accelerates the atherosclerosis in apoE-deficient mice, which also indicates the existance of the relationship between intestinal microbiota and serum cholesterol levels. Here we presume that LAB may have successfully ameliorated the intestinal microbiota disorder induced by a high-cholesterol diet and exerting a beneficial effect that influenced lipid profiles. The exact mechanism *in vivo *need be studied further to verify our presumption. Certain LAB strains have been shown to inhibit the proliferation of pathogenic bacteria [42,43]. In our study, *L. plantarum *9-41-A and *L. fermentum *M1-16 inhibited the growth of *E. coli*; in addition, the numbers of *Lactobacillus *and *Bifidobacterium *were significantly higher in the LAB-treated rats, suggesting that the two strains used can successfully tolerate gastric acid and bile salts, then benefit the proliferation of *Lactobacillus *and *Bifidobacterium *which are considered benefical to humans. Imbalances in the gut microflora could be considered to be environmental factors involved in the development of obesity and its associated metabolic disorders [44-46]. Modulation of the intestinal microbiota by supplementation with certain LAB strains may lead to body weight reduction.

By detecting the fecal water content of all rats we found that *L. plantarum *9-41-A and *L. fermentum *M1-16 increased the fecal water content in rats. Since the fecal water content can be used as an index of fecal elimination, our observations suggest that these two LAB strains have laxative potential and may stimulate bowel movements. Consequently, the transit time for cholesterol absorption in the intestine might also be reduced. This could be another explanation for the cholesterol-lowering ability of LAB strains.

Our study had some limitations, we only used Sprague-Dawley rats, and therefore, we cannot deny that the effects of LAB on cholesterol may be specific for this particular strain, and not general. So before a definite conclusion about the hypocholesterolemic effects of our lactobacilli can be reached, at least one to two more animal models require testing. Besides, Chinese people are mainly on a eastern dietaty pattern which includes wheat, rice, vegetables and fruits. Since diet can play a role in the composition and metabolic characteristics of microbiota residented in the mammalian gastrointestinal tract [47], the two strains in our present study which were isolated from the stool of healthy adult Chinese volunteers might be more suitable for the future application on Asian people who are mainly on a eastern dietaty pattern, more intensive researchs should be conducted to prove this presumption.

## Conclusions

In summary, fecal-derived *L. plantarum *9-41-A and *L. fermentum *M1-16 exerted significant hypocholesterolemic effect on Sprague-Dawley rats fed a high-cholesterol diet, with the former strain trending towards more significant reductive effects. Accordingly, the *L*.9-41-A strain resulted in more significant body weight loss and lower fat storage. The two strains are also able to contribute to a healthier intestinal microbial banlance. These findings indicate that the effects of LAB on lipid metabolism may differ among strains, and that a search for more potent and better bacterial strains was meaningful. The *L. plantarum *9-41-A strain may represent a potential therapeutic agent for controlling hyperlipidemia and lessening excessive body weight gain. The mechanisms behind these effects are likely to be diverse and will require further investigations *in vivo*.

## List of abbreviations

LAB: Lactic acid bacteria; *L*.9-41-A: *Lactobacillus plantarum *9-41-A; *L*.M1-16: *Lactobacillus fermentum *M1-16; *E. coli: Escherichia coli*; CVD: cardiovascular diseases; WHO: World Health Organization; CFU: Colony-Forming Units; WAT: white adipose tissue; HE: hematoxylin-eosin; SD: standard deviations.

## Competing interests

The authors declare that they have no competing interests.

## Authors' contributions

XN, WXH and LFG designed the study; XN, YYN, ZX, YJW, CY, WZG, FN and TY were involved in experiment conduction and analysis; XN, WXH, LXW, WCL and LFG performed data interpretation, presentation and wrote the manuscript; LXW and LFG provided significant academic advice and consultation. All authors read and approved the final manuscript.

## Pre-publication history

The pre-publication history for this paper can be accessed here:

http://www.biomedcentral.com/1472-6882/11/53/prepub
